# Dietary Patterns vs. Dietary Recommendations

**DOI:** 10.3389/fnut.2022.883806

**Published:** 2022-05-03

**Authors:** Valentina De Cosmi, Alessandra Mazzocchi, Gregorio P. Milani, Carlo Agostoni

**Affiliations:** ^1^Department of Clinical Sciences and Community Health, University of Milano, Milan, Italy; ^2^Pediatric Unit, Fondazione IRCCS Ca' Granda Ospedale Maggiore Policlinico, Milan, Italy; ^3^Pediatric Intermediate Care Unit, Fondazione IRCCS Ca' Granda Ospedale Maggiore Policlinico, Milan, Italy

**Keywords:** dietary recommendations, dietary patterns, sustainability, food preferences, complementary feeding

## Abstract

Dietary Reference Values (DRVs) are important for developing labeling laws, identifying populations at risk of over- or under-consumption, and promoting public health interventions. However, the process of developing DRVs is quite complex, and they should not be viewed as recommendations ready to use or goals for individuals. Rather, they require interpretation by professionals and can form the basis of dietary advice. On the other hand, focusing on foods rather than macronutrients can assist individuals in understanding a healthy diet by taking into consideration many variables that may help compliance with a healthy dietary style. Evolution, tradition within specific geographical and historical contexts, taste, economic affordability, season-associated local dietary resources, and lifestyle may all explain the increasing popularity of dietary patterns that are highly successful today. Three models (the Mediterranean, New Nordic, and Japanese) have been recently characterized for geographical setting and food composition, as well as the associated lifestyle. Of note, all these three models rely on pyramids sharing a large basis made up of local vegetal resources and a top of red meats (allowed in many cases, but in limited amounts), thus allowing for the urgent demand of sustainability for the planet's health. This mini-review aimed to summarize the meaning of DRVs and to describe the dietary patterns that better contemplate health, diet diversity, and sustainability.

## Introduction

### Dietary Reference Values

Dietary reference values (DRVs) are an umbrella term for a set of nutrient reference values. DRVs are the instruments for nutrition and health professionals to evaluate dietary habits and plan diets at the population level. On the one hand, these instruments allow for the identification of populations at risk of over- or under-consumption. On the other hand, they mainly refer to healthy individuals' needs since people who suffer from diseases usually have different requirements. Overall, DRVs provide the scientific basis to build nutrition recommendations and establish dietary guidelines. They are also useful for scientists involved in nutrition research and for the food industry and as they form the basis for food labeling (1).

The term “DRVs” includes the average requirement (AR), the population reference intake (PRI), the adequate intake (AI), and the reference intake range for macronutrients (RI). The AR and PRI describe the distribution of requirements in a population. These give the intake of a nutrient that meets the physiological daily needs of, respectively, half or most (97.5%) of the people in the population. Assuming normality for the distribution of the individual requirements for a nutrient, the PRI is calculated as the AR plus two times its standard deviation (SD). The AI and RI are calculated when there is insufficient scientific evidence to determine the AR and the PRI. The AI is the level of intake that is assumed to be sufficient based on observations from groups of apparently healthy people and it is interpreted as the PRI. RI indicates the range of intakes of an energy source that is adequate for maintaining health and is proposed for fat and carbohydrates based on their relative contribution to total energy intake. In the end, DRVs include a tolerable upper intake level (UL): the maximum quantity of a nutrient that can be consumed without generating adverse events over a long period of time (1). This article is a narrative mini-review that aims to summarize the meaning of DRVs, their translation into recommendations, and to characterize the dietary patterns that better contemplate health, diet diversity, and sustainability.

### Translating DRVs to Population-Based Recommendations

A healthy diet has a significant impact on health and ensuring that the population eats a healthy diet remains a public health challenge (2). As a general principle, achieving a healthy diet is possible by basing a diet on a variety of whole foods, such as fruits, vegetables, legumes, whole grains, nuts, seeds, and fish, in place of poorer quality highly processed foods. The diets that better comprehend these models are the Mediterranean, the New Nordic, and the Japanese. Each model encapsulates the culture of a population, its identity, and traditions. All of these are healthy patterns that, even with differences between each other's, since they are based on the respective local foods, share some important aspects. These patterns have in common: a great consumption of fresh fruits and nuts, vegetables, legumes (source of fiber, polyphenols, and plant proteins), cereals, and fish, and a low consumption of meats. Pyramids are visual and easy-to-understand tools for dietary guidance and nutrition education.

#### The Mediterranean Diet

The Mediterranean diet (MD) is based on the traditional foods that people used to eat in countries bordering the Mediterranean Sea. It is a dietary pattern based on the high consumption of plant-based foods, such as vegetables, whole grains, nuts, fish, and extra virgin olive oil, allowing moderate consumption of wine. It has been associated with a variety of benefits, since the 1960s, when the Seven Countries Study showed that mortality due to coronary heart diseases in the Mediterranean area was 2–3 times lower than in North Europe and the United States (3). According to different meta-analyses and large cohort studies, a two-unit increase in adherence to the MD has been associated with a reduced risk of mortality by 8, 17, and 6%. Higher levels of adherence seems associated with higher values of risk reduction (3, 4).

#### The Japanese Diet

The traditional Japanese diet (JD) has been widely considered as healthy, contributing to longevity and protecting against several non-communicable diseases (NCD) (5). A JD pyramid has been proposed by Kanauchui et al. (6). It is characterized by the moderate consumption of green tea (≥2 cups/day), at the base of the pyramid, followed by rice, miso soup, vegetables, and fruits that should be eaten every day; followed by a high consumption of fish (≥7 times/week), soy products and pickles (≥6 times/week), seaweeds, mushrooms, and Japanese-style confectionery (i.e., wagashi). At the top of the pyramid, there are meats and meat products as foods to limit.

#### The New Nordic Diet

The New Nordic diet (NND) is a dietary pattern conceived starting in 2004 and characterized by foods that are traditionally consumed and locally available in the Nordic countries. It wants to emphasize the values of potential health-promoting and gastronomic properties, sustainability, and identity of that region (7). Because of the Nordic climate, the typical foods are represented by native berries, legumes, apples, pears, root vegetables, cabbage, cauliflower, curly kale, onions, and mushrooms, as well as barley, wheat, spelt, oats, buckwheat, and rye thrive. It also implies regular fish consumption, seaweed, free-range animals and wild game (8).

#### Dietary Patterns as Cultural Models

Besides the importance of these dietary patterns in terms of health, it is worth to be highlighted their role as comprehensive cultural models that underline the importance of traditional cuisine as a means of sustainable development. In the United Nations Educational, Scientific, and Cultural Organization (UNESCO) Representative List of the Intangible Cultural Heritage of Humanity, three dietary traditions have been inscribed: the MD, the Mexican traditional cuisine, and the Washoku, traditional of the Japanese, notably for the celebration of New Year (9).

The MD, as seen in the above paragraph, is associated with health benefits, and it involves skills and traditions concerning crops, harvesting, fishing, animal husbandry, conservation, processing, and cooking. Sociality has an important role in the MD diet: eating together is the foundation of the cultural identity of communities throughout the Mediterranean basin. Shared meals represent a moment of social exchange and intercultural dialog (10).

Traditional Mexican cuisine comprehends farming, ritual practices, age-old skills, culinary techniques, and ancestral community customs and manners. The basis of traditional Mexican cuisine is the collective participation in the food chain, from planting the seeds to harvesting, to cooking and eating. The diet is founded on corn, beans, and chili and on native ingredients, such as tomatoes, squashes, avocados, cocoa, and vanilla (11).

Washoku is a Japanese social practice built on a set of skills and knowledge, strictly connected to the production, processing, preparation, and consumption of food. Respect of nature is central to this practice, and it is closely related to the sustainable use of natural resources. The Washoku characteristics are typically seen during New Year celebrations, when Japanese people make special meals that have a symbolic meaning, using beautifully decorated dishes and tableware to welcome the deities of the incoming year. Washoku favors the consumption of various natural, locally sourced ingredients, such as rice, fish, vegetables, and edible wild plants (12).

## Diet Diversity and Sustainability

An additional measure to consider when describing the value of a certain nutritional pattern is diet sustainability and diversity. Nowadays, the challenge is to prefer and follow the so-called “win-win diets,” which are dietary models built to preserve both human health and planet sustainability (13). This means nutritional patterns with evidence in the prevention and contrast of diet-related non-communicable diseases and with a positive influence on the stability of the earth's system reducing greenhouse-gas emissions, pollution, climate change, freshwater and land consumption, and biodiversity loss (14). Achieving a healthy and sustainable diet relies on preferring vegetable, organic, and minimally processed foods, as well as regional, seasonal, and fair-trade products (15). There is not a single valid green model of the food system in the world, but, in the European scenario, the MD and the NND reflect the principles of sustainable nutrition. As seen above, these nutritional patterns recommend a daily consumption of plant-based foods with a low ecological footprint. In terms of human health, the choice of this kind of products guarantees a greater supply of vitamins and minerals and other “non-nutrient” compounds, such as the fiber, which, in turn, promotes better general well-being (e.g., higher antioxidant activity, cholesterol control, and weight-body maintenance) (16). In terms of planet health, the shift from animal to plant-based foods, such as vegetables, fruits, legumes, and cereals, could reduce the environmental impact in all the different phases of the food supply chain (production, transformation, distribution, preparation, consumption, and waste management) (17). Empathizing local and seasonable products aids the regional economy as well. Considering a projected population growing to about 10 billion by 2050, the transformation of nutritional habits toward more green models is a challenging goal for the single individual, the whole population, and the next generations.

## Children's Food Preferences, Taste Experiences, and Parents' Diet

Sustainability goes hand in hand with diet diversity. The definition of diet diversity is “the number of different foods or food groups consumed over a given reference time period.” As a matter of fact, diet diversity means: seasonability, predominant vegetal over animal sources, lower emissions of greenhouse gases, and nutrition positive for the human microbiome. The predilection for plant-based, local, seasonable (and organic) foods helps to preserve the biodiversity of products, the landscapes, and the sea, and maintains the local economy (16).

Early dietary styles may be adopted from the beginning of complementary feeding (CF), a precious period in which essential nutrients must be provided to the infant and healthy dietary patterns should be established. Offering infants seasonal and local foods is a strategy to favor their acceptance and to create a habit of consumption that will last through adolescence and adult life. The recommendations from the European Society for Pediatric Gastroenterology, Hepatology, and Nutrition (ESPGHAN) state that a varied diet since the initial stages of CF should be offered to infants. Exposing infants to foods with different tastes and textures, such as bitter-tasting green vegetables, is preferred (18). Infants have an innate refusal of bitter tastes and a preference for sugar and salty foods. Families may modify these preferences doing the right choices of foods to offer. Parents' attitudes regarding the nutrition of their infants have an important role, too. It is demonstrated that responsive parenting intervention, oriented to promote the self-regulation of children, initiated in early infancy, when compared with a control intervention, results in a reduction in BMI *z*-scores at an age of 3 years (19).

**Figure 1:**
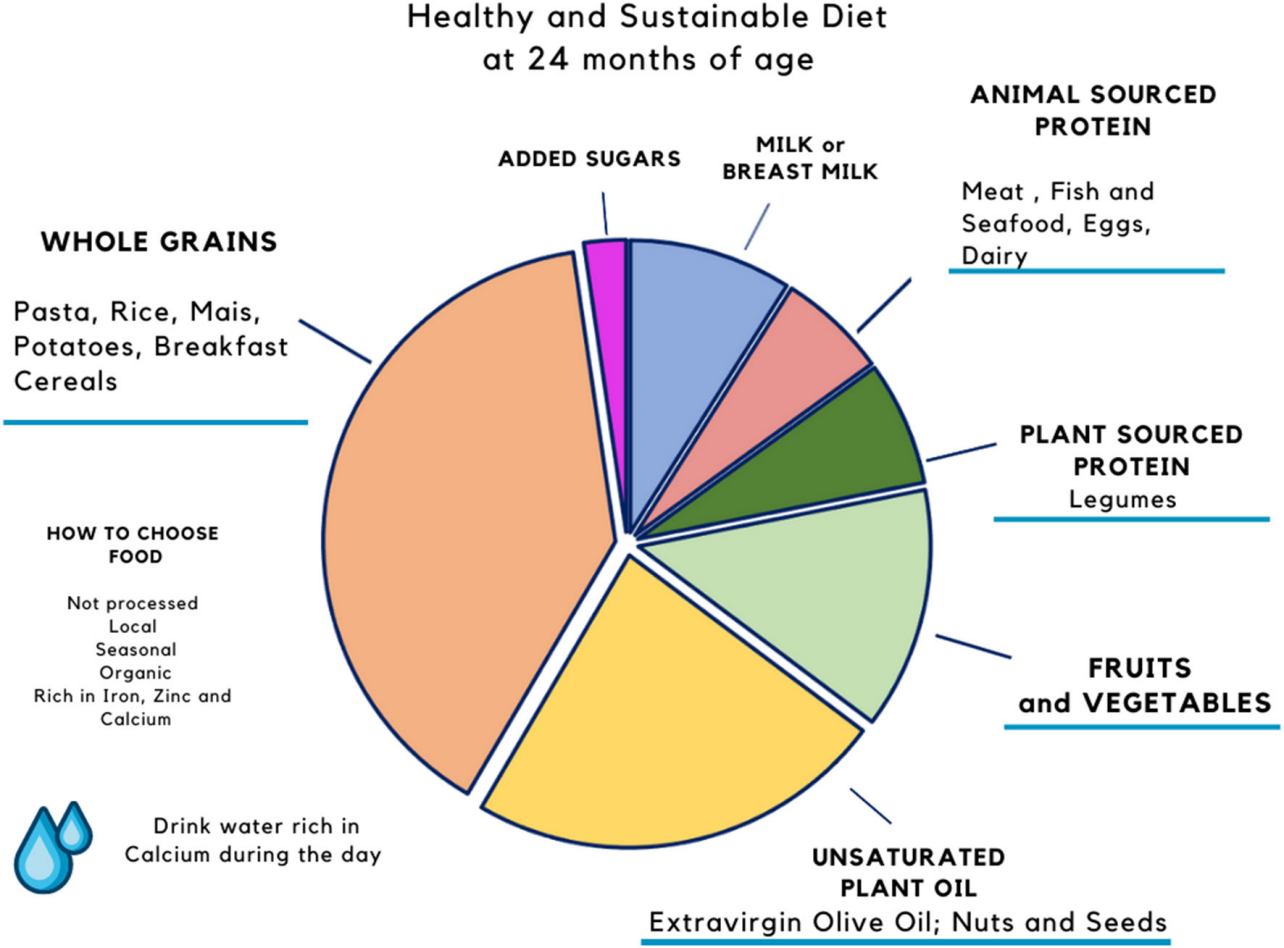
Healthy and sustainable diet at 6 months of age. Proposal of diet at 6 months of age. Percentages refer to the total amount of calories required per day by each food group for a 6-months old baby. From Mazzocchi et al. (15).

**Figure 2:**
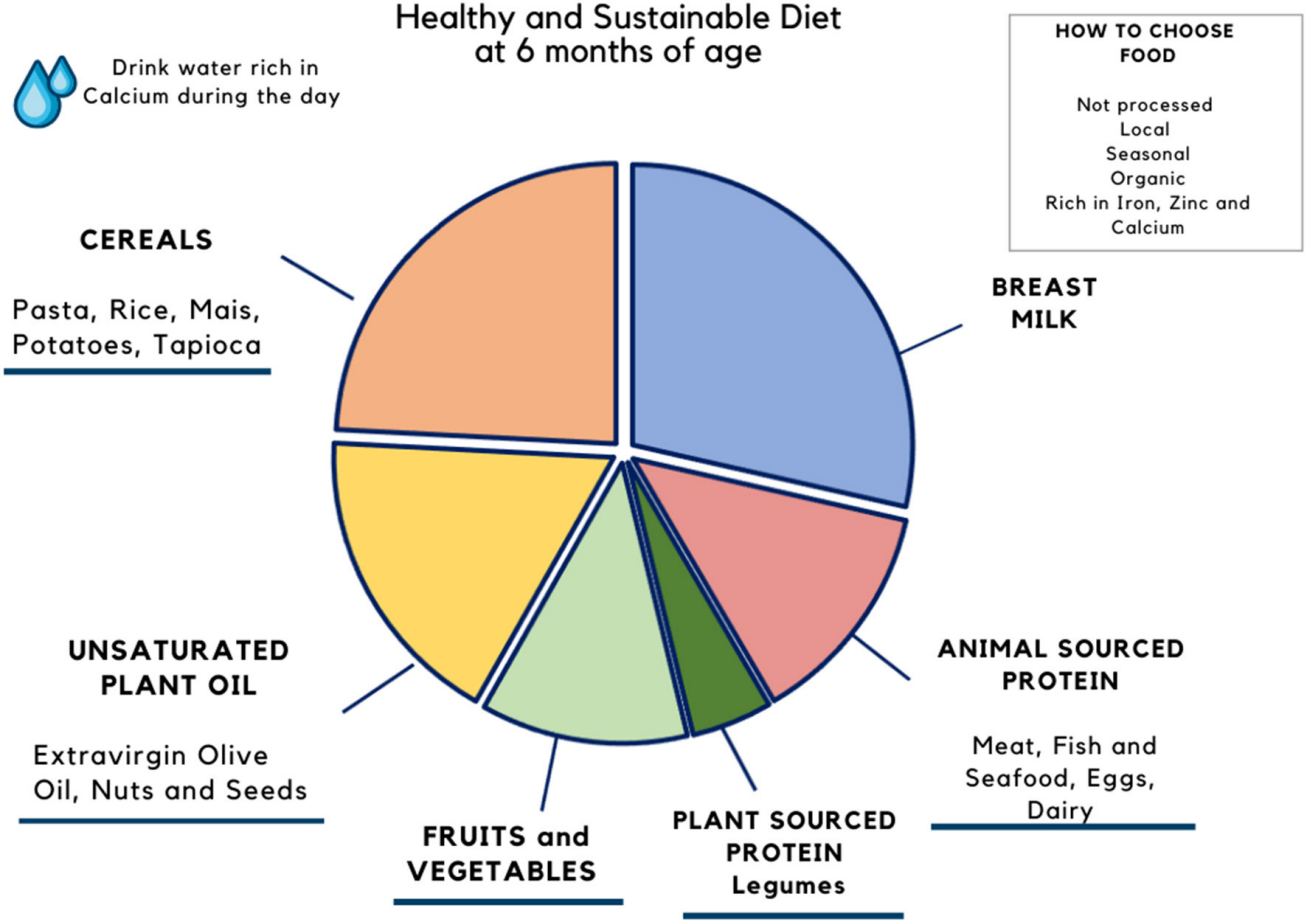
Healthy and sustainable diet at 24 months of age. Proposal of diet at 24 months of age. Percentages refer to the total amount of calories required per day by each food group for a 24-months old baby. From Mazzocchi et al. (15).

Complementary feeding is important not only for the child's growth, attitudes, and preferences but also for reorganizing the dietary choices of the whole family. It is the *window* in which new eating habits may be established, shifting the choices toward more sustainable and diverse foods, avoiding food waste. In the previous article from our group, we proposed two models of a sustainable diet that respects EFSA nutritional recommendations for an infant between 6 and 24 months of age fed breast milk on demand and 2 complimentary feeding meals (15). These models are reported in Figures 1, 2. Besides the macro- and micronutrients composition of plates, the practical advice to develop a sustainable behavior are: (1) prefer foods produced close to home, especially fruits, vegetables, and legumes; (2) choose non-processed foods, without added ingredients; (3) prefer non-packaged meals (15).

For a better understanding of the child's dietary pattern, the role of the parents' dietary pattern has been recently studied in a few reports. A study involving more than 2,500 mother–child pairs in the United States found that total fat intake was similar between maternal and child's diets (20). A survey conducted among 1,640 children identified a strong association between maternal and 3-year-old children's diet (21). Finally, a very recent study conducted in Iran observed inverse associations between mother–child dyad protein dietary intake and the risk of being underweight and wasting in children (22). These data highlight the importance of shared family dietary patterns that should be better explored in the context of the Japanese, the Mediterranean, the New Nordic, and the Mexican traditional cuisine diet models.

## Discussion

In our epoch, the two extremes of malnutrition coexist. Undernutrition and overweight, or obesity, represent both an actual burden to contrast. These two conditions can sometimes affect the same person in life: an individual who is overweight today may have had a nutritional deficit earlier in life, even in the intrauterine life. To provide healthy and sustainable dietary models, as those discussed in this article, an adequate nutritional intake, both regarding quality and quantity, especially when we consider essential nutrients important to preserve health during the life course, should be concurrently considered. This concept must be accounted for, especially in critical *windows* of life (such *windows* as infancy or the reproductive age), which are more sensitive to an optimal dietary intake (23).

Dietary patterns include sustainability (for the planet) and health (for the individual). Some dietary patterns coming around the world, particularly, we described the JD, the MD, the NND, and the Mexican Traditional cuisine as effective ways to promote a diverse and sustainable diet. From an evolutionary perspective, we may understand that “evolution-drivers” among dietary patterns exist, since nutrition evolved in different settings (23). Accordingly, foods locally selected through evolution should be the most indicated for local populations, following the season's cycles. On the other hand, the increasing availability of processed or ultra-processed food should not be ignored, and its potential effects both on health and the circular economy in the context of these models deserve new approaches and investigations (24).

This review has the strength to be a summary of dietary patterns that have both health and cultural implications. It highlights the importance of learning how to make conscious choices since infancy. By narrowing all these aspects to the complex system of personalized nutrition and sustainability, we may expect to personalize the same dietary patterns in turn. Food diversity represents the perfect connection between sustainable and personalized nutrition (25). As an example, a study aimed to challenge personalized nutrition within children following the pattern of MD, with a collaboration between Italian groups in Naples and Israel is currently ongoing. Nevertheless, some of the first researchers in the field of personalized nutrition have underlined, that even prediction models are not able to achieve a full prediction (e.g., of the glycemic response) (26) and that many confounders still need to be accounted for (27). Very few nutritional intervention trials have been planned to get long-term observations to derive useful indications. These factors have been recognized as possibly limiting the effects of personalized nutrition. Therefore, genetic heredity comes first, then the epigenetic changes through an evolutionary perspective (with the derived local lifestyle and dietary patterns), and third, the acute responses to acute changes, whose duration is highly debatable.

From a social epidemiological perspective, preserving these cultural inheritances is a way to achieve planetary health for our and future generations. In line with the present and prospective applications of personalized nutrition, future research in social interventions aimed to improve socioeconomic conditions and spread the knowledge regarding how to preserve the planetary health, at a population level, may be a target point in primary prevention.

## Conclusions

A healthy and sustainable diet is possible from infancy, but only if all aspects of the individual are concurrently considered. DRVs are important for supporting public health. Healthcare providers should help and encourage families to follow a sustainable diet from the start of CF in their children. The role of shared dietary patterns should be considered within these interventions.

We proposed two models of an ideal diet at 6 and 24 months that take into consideration both the nutritional needs of the child and the ecological footprints (15). Future studies should investigate the reliability and effectiveness of such models in the real life.

## Author Contributions

VD, AM, and GM drafted the manuscript. VD arranged the references. CA proofread the manuscript. All authors contributed significantly to the article, agreed on the manuscript in its current form, read, and agreed on the published version of the manuscript.

## Funding

This study was supported by a contribution from the Italian Ministry of Health (IRCCS Grant).

## Conflict of Interest

The authors declare that the research was conducted in the absence of any commercial or financial relationships that could be construed as a potential conflict of interest.

## Publisher's Note

All claims expressed in this article are solely those of the authors and do not necessarily represent those of their affiliated organizations, or those of the publisher, the editors and the reviewers. Any product that may be evaluated in this article, or claim that may be made by its manufacturer, is not guaranteed or endorsed by the publisher.

## References

[1] EFSA EFSA Panel on Dietetic Products N Allergies. scientific opinion on principles for deriving and applying dietary reference values. EFSA J. (2010) 8:1458. 10.2903/j.efsa.2010.1458

[2] Milani GP Silano M Mazzocchi A Bettocchi S De Cosmi V Agostoni C. Personalized nutrition approach in pediatrics: a narrative review. Pediatr Res. (2021) 89:384–8. 10.1038/s41390-020-01291-8 33230198

[3] Dontas AS Zerefos NS Panagiotakos DB Vlachou C Valis DA. Mediterranean diet and prevention of coronary heart disease in the elderly. Clin Interv Aging. (2007) 2:109–15. 10.2147/ciia.2007.2.1.109 18044083 PMC2684076

[4] Minelli P Montinari MR. The Mediterranean Diet And Cardioprotection: Historical Overview And Current Research. J Multidiscip Healthc. (2019) 12:805–15. 10.2147/JMDH.S219875 31632049 PMC6776290

[5] Gabriel AS Ninomiya K Uneyama H. The role of the Japanese traditional diet in healthy and sustainable dietary patterns around the world. Nutrients. (2018) 10:173. 10.3390/nu10020173 29401650 PMC5852749

[6] Kanauchi M Kanauchi K. Proposal for an empirical Japanese diet score and the Japanese diet pyramid. Nutrients. (2019) 11:2741. 10.3390/nu11112741 31726696 PMC6893777

[7] Agnihotri N Rudjord Hillesund E Bere E Wills AK Brantsaeter AL Øverby NC. Development and description of New Nordic Diet scores across infancy and childhood in the Norwegian mother, father and child cohort study (MoBa). Matern Child Nutr. (2021) 17:e13150. 10.1111/mcn.13150 33528109 PMC8189223

[8] Mithril C Dragsted LO Meyer C Tetens I Biltoft-Jensen A Astrup A. Dietary composition and nutrient content of the New Nordic Diet. Public Health Nutr. (2013) 16:777–85. 10.1017/S1368980012004521 23089239 PMC10271429

[9] Intangible Cultural Heritage - UNESCO Washoku Traditional Traditional Dietary Cultures of the Japanese Notably Notably for the Celebration of New Year. Available online at: https://ich.unesco.org/en/RL/washoku-traditional-dietary-cultures-of-the-japanese-notably-for-the-celebration-of-new-year-00869. (accessed on March 23, 2022).

[10] Intangible, Cultural Heritage - UNESCO Mediterranean Diet,. Available online at: https://ich.unesco.org/en/RL/mediterranean-diet-00884 (accessed on March 23, 2022).

[11] Intangible Cultural Heritage - UNESCO Traditional Mexican Cuisine - Ancestral Ongoing Community Culture the Michoacán Paradigm. Available online at: https://ich.unesco.org/en/RL/traditional-mexican-cuisine-ancestral-ongoing-community-culture-the-michoacn-paradigm-00400. (accessed on March 23, 2022).

[12] Yatsuya H Tsugane S. What constitutes healthiness of Washoku or Japanese diet? Eur J Clin Nutr. (2021) 75:863–4. 10.1038/s41430-021-00872-y 33603149 PMC7890542

[13] Willett W Rockström J Loken B Springmann M Lang T Vermeulen S. Food in the anthropocene: the EAT-lancet commission on healthy diets from sustainable food systems. Lancet. (2019) 393:447–92. 10.1016/S0140-6736(18)31788-4 30660336

[14] Hachem F Vanham D Moreno LA. Territorial and sustainable healthy diets. Food Nutr Bull. (2020) 41(Suppl. 2):87s−103s. 10.1177/0379572120976253 33356591

[15] Mazzocchi A De Cosmi V Scaglioni S Agostoni C. Towards a more sustainable nutrition: complementary feeding and early taste experiences as a basis for future food choices. Nutrients. (2021) 13:2695. 10.3390/nu13082695 34444855 PMC8398974

[16] Serra-Majem L Tomaino L Dernini S Berry EM Lairon D Ngo de la Cruz J. Updating the mediterranean diet pyramid towards sustainability: focus on environmental concerns. Int J Environ Res Public Health. (2020) 17:8758. 10.3390/ijerph17238758 33255721 PMC7728084

[17] von Koerber K Bader N Leitzmann C. Wholesome Nutrition: an example for a sustainable diet. Proc Nutr Soc. (2017) 76:34–41. 10.1017/S0029665116000616 27502053

[18] Fewtrell M Bronsky J Campoy C Domellöf M Embleton N Fidler Mis N. Complementary feeding: a position paper by the european society for paediatric gastroenterology, hepatology, and nutrition (ESPGHAN) committee on nutrition. J Pediatr Gastroenterol Nutr. (2017) 64:119–32. 10.1097/MPG.0000000000001454 28027215

[19] Paul IM Savage JS Anzman-Frasca S Marini ME Beiler JS Hess LB. Effect of a responsive parenting educational intervention on childhood weight outcomes at 3 years of age: the INSIGHT randomized clinical trial. JAMA. (2018) 320:461–8. 10.1001/jama.2018.9432 30088009 PMC6142990

[20] Beydoun MA Wang Y. Parent-child dietary intake resemblance in the United States: evidence from a large representative survey. Soc Sci Med. (2009) 68:2137–44. 10.1016/j.socscimed.2009.03.029 19375837 PMC2730650

[21] Fisk CM Crozier SR Inskip HM Godfrey KM Cooper C Robinson SM. Influences on the quality of young children's diets: the importance of maternal food choices. Br J Nutr. (2011) 105:287–96. 10.1017/S0007114510003302 20807465

[22] Moradi M Jalilpiran Y Askari M Surkan PJ Azadbakht L. Associations between mother-child dyad dietary patterns and child anthropometric measures among 6-year-old children. Eur J Pediatr. (2022) 181:225–34. 10.1007/s00431-021-04180-2 34259893

[23] Wells JC Sawaya AL Wibaek R Mwangome M Poullas MS Yajnik CS. The double burden of malnutrition: aetiological pathways and consequences for health. Lancet. (2020) 395:75–88. 10.1016/S0140-6736(19)32472-9 31852605 PMC7613491

[24] Capozzi F Magkos F Fava F Milani GP Agostoni C Astrup A. A multidisciplinary perspective of ultra-processed foods and associated food processing technologies: a view of the sustainable road ahead. Nutrients. (2021) 13:3948. 10.3390/nu13113948 34836203 PMC8619086

[25] Agostoni C Boccia S Banni S Mannucci PM Astrup A. Sustainable and personalized nutrition: from earth health to public health. Eur J Intern Med. (2021) 86:12–6. 10.1016/j.ejim.2021.02.012 33640245

[26] Zeevi D Korem T Zmora N Israeli D Rothschild D Weinberger A. Personalized nutrition by prediction of glycemic responses. Cell. (2015) 163:1079–94. 10.1016/j.cell.2015.11.001 26590418

[27] Bashiardes S Abdeen SK Elinav E. Personalized nutrition: are we there yet? J Pediatr Gastroenterol Nutr. (2019) 69:633–8. 10.1097/MPG.0000000000002491 31765333

